# Hormone use for menopausal symptoms and risk of breast cancer. A Danish cohort study

**DOI:** 10.1038/sj.bjc.6602472

**Published:** 2005-03-22

**Authors:** M Ewertz, L Mellemkjaer, A H Poulsen, S Friis, H T Sørensen, L Pedersen, J K McLaughlin, J H Olsen

**Affiliations:** 1 Department of Oncology, Aalborg Hospital, Aarhus University, Hobrovej 18-22, PO Box 365, DK-9100 Aalborg, Denmark; 2 Institute of Cancer Epidemiology, Danish Cancer Society, Strandboulevarden 49, DK-2100 Copenhagen, Denmark; 3 Department of Clinical Epidemiology, Aarhus University, Vennelyst Boulevard 6, DK-8000 Aarhus, Denmark; 4 Vanderbilt University Medical Center, Vanderbilt-Ingram Cancer Center, Nashville, TN, USA; 5 International Epidemiology Institute, Rockville, MD, USA

**Keywords:** hormone replacement therapy, breast cancer, population-based study

## Abstract

Numerous studies and meta-analyses have shown that hormone replacement therapy (HRT) for menopausal symptoms increases the risk of developing breast cancer, estimated to be 2.3% for each year of use. The influence of different oestrogen–progestin regimens has still not been fully evaluated. Using longitudinal data from the population-based prescription database of the county of North Jutland, Denmark, and the Danish Cancer Registry, we examined the risk of developing breast cancer in relation to HRT in a cohort of 78 380 women aged 40–67 years from 1989 to 2002. A total of 1462 cases of breast cancer were identified during a mean follow-up of 10 years. Use of HRT did not increase the risk of breast cancer in women aged 40–49 years. Restricting the cohort to 48 812 women aged 50 years or more at entry, of whom 15 631 were HRT users, we found an increased risk associated with current use of HRT (relative risk 1.61, 95% confidence interval 1.38–1.88). The risk increased with increasing duration of use and decreased with time since last HRT prescription, reaching unity after 5 years. No material risk difference was observed among the various HRT-regimens. This population-based cohort study provides further confirmation that HRT increases the risk of developing breast cancer in women aged 50 years or more.

Numerous studies have shown that hormone replacement therapy (HRT) with oestrogen with or without progestin for menopausal symptoms increases the risk of breast cancer. A collaborative reanalysis of data from 51 epidemiological studies of 52 705 women with breast cancer and 108 411 women without breast cancer demonstrated a 2.3% increase in breast cancer risk for each year of HRT use (Collaborative Group on Hormonal Factors in Breast Cancer, 1997). The highest risk estimates were seen for current and recent use of long duration, with the relative risk (RR) for 5 or more years of use being 1.35 (95% confidence interval (CI) 1.21–1.49). More recent studies from Denmark (Tjønneland *et al*, 2004) and the UK (Million Women Study, 2003) reported RR estimates for current HRT use of 2.22 (95% CI 1.80–2.75) and 1.66 (95% CI 1.58–1.75), with evidence of lower breast cancer risk for oestrogen-only HRT compared to combined oestrogen–progestin HRT from these and other observational studies (Magnusson *et al*, 1999; Stahlberg *et al*, 2004). US randomised controlled trials have reported RR estimates of 1.30 (95% CI 0.77–2.19) and 1.26 (95% CI 1.00–1.59) for combined HRT and 0.77 (95% CI 0.59–1.00) for unopposed oestrogen (Hulley and Grady, 2004; Women's Health Initiative, 2002, 2004). Risk estimates tended to increase with increasing duration of HRT use and to decrease with time since last exposure.

We have conducted a study on HRT use in relation to the risk of breast cancer using the population-based prescription database of North Jutland, Denmark, which allows for a complete non-self reported history of HRT exposure, including the specific type, to be derived for every woman in the county.

## MATERIAL AND METHODS

The study was conducted within the population of North Jutland, a county with nearly 500 000 inhabitants, representing approximately 9% of the total Danish population. The National Health Service in Denmark provides tax supported health care for all inhabitants, guaranteeing free access to general practitioners, hospitals and public clinics, and refunds a variable proportion of the costs of medication prescribed by physicians through a computerised accounting system. In North Jutland, this accounting system also provides prescription data to the Pharmaco-Epidemiologic Prescription Database, which was initiated in 1989 (Gaist *et al*, 1997) and by 1991 covered all pharmacies in the county. The Database includes the civil personal registration number (a unique number assigned to all Danish residents that encodes gender and date of birth) of the patient, type of drug prescribed according to Anatomical Therapeutical Chemical (ATC) Classification System (Capellá, 1993) and the date of prescription (date of dispensing the drug). The civil personal identification number is maintained by the Central Population Register (CPR), which updates information on vital status (dates of death or emigration), address (date of migration from the county), and the civil personal identification numbers of all offspring. Thus, information on parity can be obtained for all women since the establishment of the CPR in 1968.

From the files of the CPR, we identified 83 873 women who were 40–66 years of age at any time during the period 1 January 1989 to 31 December 2002 and resident in the county of North Jutland. These women were linked to the Danish Cancer Registry to identify cases of breast cancer occurring through 2002. The Danish Cancer Registry has recorded incident cases of cancer on a nation-wide basis since 1943 with accurate and virtually complete ascertainment (Storm *et al*, 1997). Tumours are classified according to a revised version of ICD-7 (Danish National Board of Health, 2003), and since 1978, also according to ICD-O (WHO, 1976). We excluded 1444 women who had a cancer diagnosis (except nonmelanoma skin cancer) before 1989 or before age 40 (if later than 1989).

The remaining 82 429 women were linked to the Pharmaco-Epidemiologic Prescription Database. We excluded 1079 women who received prescriptions for sex hormones other than those used in HRT (ATC codes: G03B, G03G, G03H, G03X) including androgens, during 1989–2002, and 2970 women who had used systemic HRT before the age of 40 years. Among the remaining 78 380 women, we identified 17 466 who received at least two prescriptions for systemic HRT (G03A, G03C, G03D and G03F) from 1 January 1989 until 31 December 2002. In Denmark, there is normally no reimbursement for oral contraceptives, but we included women recorded with reimbursed prescriptions for oral contraceptives, since this indicates that the hormones were given for reasons other than contraception. Prescription of nonsystemic HRT was not judged as HRT exposure for the purposes of the present study.

**Figure 1:**
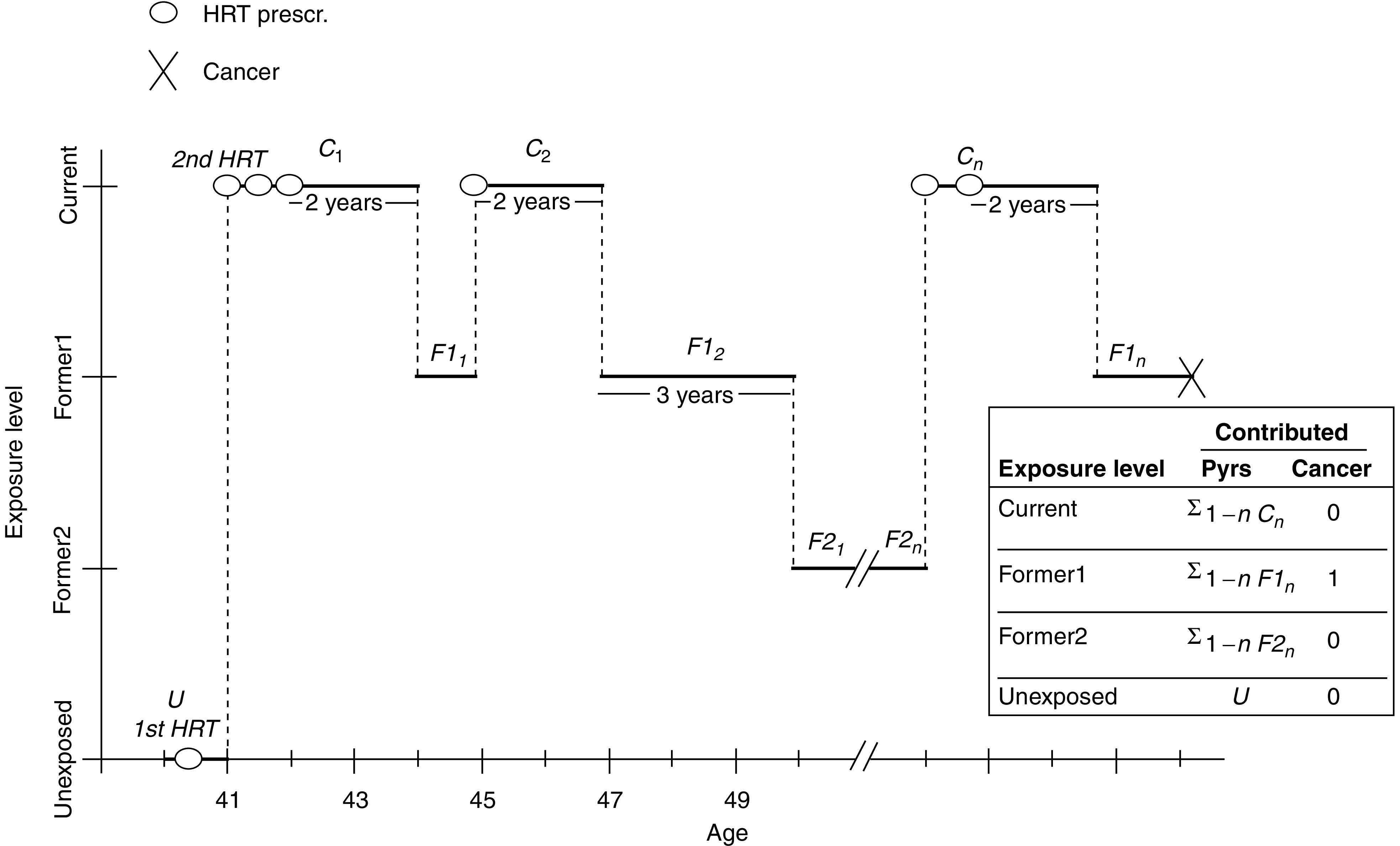
Illustration of the method of calculation of exposure to HRT in the cohort of women aged 40–67 years in North Jutland county, Denmark, 1989–2002.

The follow-up for breast cancer started on 1 January 1989 or at age 40 years, whichever occurred later, and continued until the date of breast cancer diagnosis, of cancer other than breast (except) for nonmelanoma skin cancer), date of death, of migration from North Jutland, or 31 December 2002, whichever came first. Since women with only one prescription may never have actually taken the drug, we classified such women as nonexposed. The follow-up time was stratified according to use of HRT in unexposed time (less than two prescriptions) and exposed time (two or more prescriptions) (Figure 1). The exposed time was further stratified into:

1. recency of use (current use of HRT with less than 2 years since last prescription, recent use with 2–5 years since last prescription and former use with more than 5 years since last prescription);
2. number of prescriptions (2–4, 5–9, 10–19, and 20 or more prescriptions);
3. type of first prescription of HRT (oestrogen only, sequentially combined oestrogen–testosterone derived progestin (*levonorgestrel*, *norethisteron*, *norgestimat*, *desogestrel*, *gestoden*), sequentially combined oestrogen–progesterone derived progestin (*medroxyprogesteron*), continuously combined oestrogen–testosterone derived progestin (*norethisteron*), tibolone, and progestins. Users contributed person-years to the appropriate group until they received a prescription for HRT from one of the other groups, and from that time on they contributed person-years to a category of mixed use.

We computed rate ratios as the breast cancer incidence rate for HRT exposure divided by the breast cancer incidence rate for nonexposure (<two prescriptions). Age-specific as well as age-standardised (direct standardisation in 5-year age-groups to the age distribution in the total cohort of women) rate ratios were calculated. In addition, we performed analyses based on Cox proportional hazards models with age as the time scale. These analyses were adjusted for calendar-period as a time-dependent covariate with two levels (1989–1996 and 1997–2002) and number of children and age at first child obtained from the CPR as time-dependent linear variables. Tests were based on the likelihood ratio test statistics calculated from Cox's partial likelihood. Confidence intervals were based on Wald's test of the corresponding regression parameters, that is, on the log scale for the rate ratios. The statistical analyses were performed using SAS version 8.02. The proportional hazards assumption of the Cox models was tested by visual inspection of transformations of the survival function using standard techniques.

## RESULTS

In the cohort of 78 380 women we identified 1462 cases of breast cancer during a mean follow-up of 10 years (maximum 14 years). Other details of the cohort including year of entry, number of children, and age at first birth are presented in Table 1.

Table 2 shows breast cancer incidence among women exposed and unexposed to HRT by age. In comparison with the unexposed, breast cancer incidence was nonsignificantly lower among those exposed in the age group 40–49 years, with a relative risk (RR) of 0.56 and 0.88 for ages 40–44 and 45–49, respectively. In contrast, for those over 50 years of age RRs were generally significantly elevated, ranging from 1.19 for ages 50–54 to 3.71 for ages 65–67.

Women aged 40–49 years are likely to represent a mixture of pre-, peri-, and postmenopausal women and may have used HRT for a variety of reasons, not only for menopausal symptoms. To study HRT use mainly in postmenopausal women and having no information on menopausal status, we restricted the subsequent analyses to the person-time contributed by women after they had reached 50 years of age. With this restriction, the cohort was reduced to 48 812 women with 869 breast cancers during a mean follow-up of 7.6 years (Table 1). However, we made use of the HRT exposure information available from age 40–49 years so that, for instance, a 50-year-old woman who had filled six prescriptions from age 47 to 49 entered the cohort at this exposure level (see Figure 1).

Of the 48 812 women aged 50 years or more, 15 631 or 32% had filled two or more prescriptions for HRT. Table 3 shows that close to half (47%) of these women began using HRT before age 50, that more than 60% of users filled more than 10 prescriptions, and that the most commonly used type of HRT was the sequential combination of oestrogen and a progestin.

Among women age 50 or older, current use of HRT was associated with a significantly increased risk of breast cancer, with an RR of 1.61 (95% CI 1.38–1.88), adjusted for calendar period, number of children and age at first birth (Table 4). The risk increased with increasing duration of use estimated by the number of prescriptions filled, with RR=1.86 (95% CI 1.54–2.25) for 20 or more prescriptions, and decreased with time since last HRT prescription, returning to baseline after 5 or more years.

Elevated risks were observed for oestrogens alone, progestins alone, mixed use, and sequential preparations of oestrogen and testosterone derived progestin, while the risk was not increased for sequential preparations of oestrogen and progesterone derived progestin, continuous combined therapy, and for tibolone. However, each of the latter estimates was based on fewer than 15 cases. A total of 790 women had at least one prescription for tibolone, but since tibolone is mostly prescribed when more conventional HRT is tolerated poorly, tibolone use is therefore generally included in the category of mixed use. When the category of mixed use was split into those with at least one prescription for tibolone (eight breast cancers in 2492 person-years) and those without tibolone (132 breast cancers in 40 894 person-years), adjusted RR estimates were 1.37 (95% CI 0.68–2.75) and 1.51 (95% CI 1.25–1.84) respectively.

## DISCUSSION

The breast cancer findings of the present population-based cohort study are consistent with those of prior observational studies of HRT (Collaborative Group on Hormonal Factors in Breast Cancer, 1997; Million Women Study, 2003; Tjønneland *et al*, 2004). RR estimates from randomised controlled trials tend to be lower than those from observational studies for both combined and unopposed HRT (Hulley and Grady, 2004). If, as indicated by the Women's Health Initiative (2004), unopposed HRT does not increase the risk of breast cancer, then the increased RR estimate for unopposed oestrogen in the present study may reflect uncontrolled bias inherent in observational studies of HRT. Similar bias should be present for all HRT regimens, and thus it is noteworthy that no material difference was observed among RR estimates for the various HRT regimens.

The strength of this study is that it was conducted in a well-defined geographical area for which standardised population-based information is available on both exposure and disease. The data on exposure provide a full history of prescriptions for HRT during follow-up, including specific information on HRT formulation. We are confident the exposure information is reasonably complete since it is very unusual for the study population to consult doctors or purchase prescription drugs outside their own county, and HRT is available in Denmark by prescription only. In a study of validity of self-reported use of HRT 95% of Danish nurses registered in the prescription database as being prescribed HRT, reported having complied with the treatment (Løkkegaard *et al*, 2004). Breast cancer ascertainment can also be regarded as almost complete (Storm *et al*, 1997). We were able to control for potentially confounding effects of number of children and age at first birth through complete registry-based information on these variables. Social status might also be considered to be a potential confounder since breast cancer is associated with high socioeconomic status (Danø *et al*, 2003), but in Denmark no substantial socioeconomic gradient in HRT use has been detected (Olesen *et al*, 2004).

Limitations of our study include the lack of information on such potentially confounding factors as age at menopause and body mass index (BMI). Overall, among women aged 40–49 years, we did not detect an increased breast cancer risk associated with HRT use, which may well be due to the fact that these women represent a mixture of pre-, peri-, and postmenopausal states. Despite the lack of control for age at menopause, our risk estimate of RR=1.61 (95% CI 1.38–1.88) for current HRT use in women aged 50 years or more agrees well with that reported for postmenopausal women from the Million Women Study (2003) (RR=1.66 (95% CI 1.58–1.75)). Several studies have found an interaction between HRT use and BMI, the risk associated with HRT (Huang *et al*, 1997; Million Women Study, 2003) being higher in lean than in overweight women. Our study cannot address the issue of interaction between HRT use and BMI, and our inability to control for BMI may have led to underestimation of risks associated with HRT use.

With regard to the different types of HRT, our results were similar to those reported in the Million Women Study and a recent Danish study (Stahlberg *et al*, 2004) for oestrogen only and for less than 5 years use of sequential preparations of oestrogen and testosterone derived progestin. The risk associated with use of tibolone was not materially different from other types of HRT. Our findings of no increased risk for sequential preparations of oestrogen and progesterone derived progestin and continuous combined therapy are somewhat surprising, but since these estimates were based on fewer than 15 cases, chance cannot be ruled out as an explanation.

In conclusion, this study reports elevated risk estimates for breast cancer with HRT use in women over age 50 years, increasing with duration of use, and with no evidence of increased risk 5 years after stopping HRT. Risk did not differ markedly for treatment with oestrogen alone, progestins alone, or combinations thereof.

## Figures and Tables

**Table 1:** Characteristics of the study cohort of Danish women by age at entry

	**Follow-up starting** **at 40 years**	**Follow-up starting** **at 50 years**
Number of women	78 380	48 812
Cases of breast cancer	1462	869
Mean years of follow-up	10.0	7.6
		
*Year of entry into cohort*		
1989	42 655	10 241
1990	3135	2541
1991	2997	2677
1992	2975	2912
1993	2882	3128
1994	2815	3208
1995	2826	3300
1996	2734	3365
1997	2749	3242
1998	2612	3046
1999	2567	2848
2000	2564	2857
2001	2464	2753
2002	2405	2694
		
*Number of children*		
0	7148	3928
1	9976	5907
2	35 682	21 662
3	18 754	12 385
4–5	6374	4586
6+	446	344
		
*Age at first birth*		
10–14	24	12
15–19	12 664	9027
20–24	34 613	22 706
25–29	18 045	10 244
30–34	4541	2225
35–39	1220	590
40+	125	80

**Table 2:** Age-specific breast cancer incidence rates per 100 000 woman-years among HRT exposed and unexposed women, and rate ratios (RR) for HRT-exposure, North Jutland county, Denmark, 1989–2002

**Age (years)**	**HRT exposed**			**Unexposed**			**RR**	**95% CI**
	**Person-years**	**No. of cases**	**Rate**	**Person-years**	**No. of cases**	**Rate**		
40–44	3427	2	58.4	179 258	188	104.9	0.56	0.07–2.01
45–49	21 908	37	168.9	191 762	366	190.9	0.88	0.62–1.22
50–54	44 163	90	203.8	150 164	257	171.1	1.19	0.96–1.46
55–59	41 022	123	299.8	89 173	193	216.4	1.39	1.15–1.65
60–64	17 453	78	446.9	38 427	99	257.6	1.73	1.37–2.17
65–67	1725	17	985.6	4513	12	265.9	3.71	2.16–5.94

**Table 3:** Characteristics of HRT users aged 50–67 years in North Jutland county, Denmark, 1989–2002

	**No. of women**	**%**
Total	15 631	100.0
*Year of second HRT-prescription*		
1989–1990	3676	23.5
1991–1992	2540	16.2
1993–1994	2784	17.8
1995–1996	2140	13.7
1997–1998	1799	11.5
1999–2000	1439	9.2
2001–2002	1255	8.0
		
*Age at second HRT-prescription*		
40–44	1460	9.3
45–49	5829	37.3
50–54	6971	44.6
55–59	1210	7.7
60–64	150	1.0
65–66	11	0.1
		
*Number of HRT-prescriptions*		
2–4	3327	21.3
5–9	2493	15.9
10–19	2994	19.2
20–39	4084	26.1
40–59	2230	14.3
60+	503	3.2
		
*Type of HRT* a		
Oestrogen only	2965	19.0
Sequential oestrogen–testosterone-derived progestin^b^	5652	36.2
Sequential oestrogen–progesterone-derived progestin^c^	1221	7.8
Continuous oestrogen–testosterone-derived progestin^d^	1356	8.7
Tibolone	97	0.6
Gestagenes only	1918	12.3
Mixed use	2422	15.5

a Determined from first and second prescription ever.

b Testosterone-derived refers to levonorgestrel, norethisteron, norgestimat, desogestrel, gestoden.

c Progesterone-derived refers to medroxyprogesteron.

d Testosterone-derived refers to norethisteron.

**Table 4:** Breast cancer risk associated with HRT-use among women aged 50–67 in North Jutland county, Denmark, 1989–2002

	**No. of cases**	**Person-years**	**Age-stnd. rate^a^**	**SRR^b^**	**Min. adj. RR^c^**	**95% CI**	**Fully adj. RR^d^**	**95% CI**
Unexposed	561	282.278	200.4	Ref.	Ref.		Ref.	
								
*HRT*								
2+ prescriptions	308	104.362	283.9	1.42	1.39	1.21–1.60	1.40	1.22–1.61
								
*Recency of HRT-use* d								
Current use (<2 years since last prescription)	222	67.224	331.8	1.66	1.60	1.37–1.88	1.61	1.38–1.88
Former use (2–5 years since last prescription)	55	22.540	243.6	1.22	1.13	0.85–1.49	1.15	0.87–1.51
Former use (5+ years since last prescription)	31	14.598	212.4	0.77	0.87	0.61–1.23	0.89	0.62–1.28
								
*No. of HRT-prescriptions*								
2–4	52	24.262	215.5	1.08	1.06	0.80–1.41	1.08	0.81–1.43
5–9	46	20.471	232.3	1.16	1.12	0.83–1.52	1.13	0.84–1.53
10–19	63	25.184	248.8	1.24	1.23	0.95–1.59	1.23	0.95–1.60
20+	147	34.445	348.7	1.74	1.86	1.54–2.24	1.86	1.54–2.25
								
*HRT-type* e								
Oestrogen only	50	17.888	260.4	1.30	1.29	0.97–1.73	1.35	1.01–1.80
Sequential oestrogen–testost.-derived progestin	80	25.740	317.8	1.59	1.53	1.21–1.93	1.52	1.21–1.93
Sequential oestrogen–progest.-derived progestin	6	5.062	104.2	0.52	0.58	0.26–1.29	0.57	0.26–1.28
Continuous oestrogen–testost.-derived progestin	13	5.851	224.0	1.12	0.99	0.57–1.72	0.99	0.57–1.72
Tibolone	1	509	141.4	0.71	0.86	0.12–6.14	0.84	0.12–5.95
Progestins only	18	6.441	305.5	1.52	1.42	0.89–2.28	1.36	0.87–2.24
Mixed use	140	42.871	307.5	1.53	1.50	1.24–1.36	1.51	1.25–1.82

a Incidence per 100 000 person-years. Direct standardisation to studybase.

b Standardised rate ratio.

c Cox proportional hazards model, adjusted for calendar period.

d Cox proportional hazards model, adjusted for calendar period and number of children and age at first birth.

e HRT-type is determined from 1st prescription, a prescription from any other group causes transfer to mixed use group.
